# Towards the Search for Potential Biomarkers in Osteosarcoma: State-of-the-Art and Translational Expectations

**DOI:** 10.3390/ijms232314939

**Published:** 2022-11-29

**Authors:** Leonel Pekarek, Basilio De la Torre-Escuredo, Oscar Fraile-Martinez, Cielo García-Montero, Miguel A. Saez, David Cobo-Prieto, Luis G. Guijarro, Jose V. Saz, Patricia De Castro-Martinez, Diego Torres-Carranza, Tatiana Pekarek, Ana Clara Carrera, Melchor Alvarez-Mon, Miguel A. Ortega

**Affiliations:** 1 Department of Medicine and Medical Specialities, Faculty of Medicine and Health Sciences, University of Alcalá, 28801 Alcala de Henares, Spain; 2 Ramón y Cajal Institute of Sanitary Research (IRYCIS), 28034 Madrid, Spain; 3 Oncology Service, Guadalajara University Hospital, 19002 Guadalajara, Spain; 4 Department of Surgery, Medical and Social Sciences, Faculty of Medicine and Health Sciences, University of Alcalá, 28801 Alcala de Henares, Spain; 5 Service of Traumatology, University Hospital Ramón y Cajal, 28034 Madrid, Spain; 6 Pathological Anatomy Service, Central University Hospital of Defence-UAH Madrid, 28801 Alcala de Henares, Spain; 7 Immune System Diseases-Rheumatology Service, Central University Hospital of Defence-UAH Madrid, 28801 Alcala de Henares, Spain; 8 Unit of Biochemistry and Molecular Biology, Department of System Biology (CIBEREHD), University of Alcalá, 28801 Alcala de Henares, Spain; 9 Department of Biomedicine and Biotechnology, Faculty of Medicine and Health Sciences, University of Alcalá, 28801 Alcala de Henares, Spain; 10 Department of Immunology and Oncology, Centro Nacional de Biotecnología, Consejo Superior de Investigaciones Científicas (CSIC), Universidad Autónoma de Madrid, 28049 Madrid, Spain; 11 Immune System Diseases-Rheumatology, Oncology Service an Internal Medicine (CIBEREHD), University Hospital Príncipe de Asturias, 28806 Alcala de Henares, Spain; 12 Cancer Registry and Pathology Department, Principe de Asturias University Hospital, 28806 Alcala de Henares, Spain

**Keywords:** osteosarcoma, microRNA, genetic markers, serological markers, circulating tumour cells, immunohistochemical markers

## Abstract

Osteosarcoma represents a rare cause of cancer in the general population, accounting for <1% of malignant neoplasms globally. Nonetheless, it represents the main cause of malignant bone neoplasm in children, adolescents and young adults under 20 years of age. It also presents another peak of incidence in people over 50 years of age and is associated with rheumatic diseases. Numerous environmental risk factors, such as bone diseases, genetics and a history of previous neoplasms, have been widely described in the literature, which allows monitoring a certain group of patients. Diagnosis requires numerous imaging tests that make it possible to stratify both the local involvement of the disease and its distant spread, which ominously determines the prognosis. Thanks to various clinical trials, the usefulness of different chemotherapy regimens, radiotherapy and surgical techniques with radical intent has now been demonstrated; these represent improvements in both prognosis and therapeutic approaches. Osteosarcoma patients should be evaluated in reference centres by multidisciplinary committees with extensive experience in proper management. Although numerous genetic and rheumatological diseases and risk factors have been described, the use of serological, genetic or other biomarkers has been limited in clinical practice compared to other neoplasms. This limits both the initial follow-up of these patients and screening in populations at risk. In addition, we cannot forget that the diagnosis is mainly based on the direct biopsy of the lesion and imaging tests, which illustrates the need to study new diagnostic alternatives. Therefore, the purpose of this study is to review the natural history of the disease and describe the main biomarkers, explaining their clinical uses, prognosis and limitations.

## 1. Introduction

Among neoplasms, sarcomas represent a heterogeneous group of malignant neoplasms of mesenchymal origin that comprises a wide variety of histological subgroups; each malignancy can manifest in any anatomical location and carries a complexity of diagnosis and prognosis. Overall, more than 80% of sarcomas correspond to soft tissues (mainly liposarcomas, leiomyosarcomas and undifferentiated sarcomas), while 20% correspond to bone, with osteosarcoma being the most frequent primary malignant tumour, followed by Ewing’s sarcoma and chondrosarcoma, among others [1]. We must mention that there are more than 100 histological subtypes in both soft tissue sarcomas and bone sarcomas, requiring a complex diagnosis. As a whole, sarcomas are rare neoplasms accounting for less than 1% of malignant neoplasms, which are diagnosed at a rate of approximately 3.4 cases per million inhabitants worldwide [2]. In Spain, for example, in 2021, 141 deaths from malignant tumours of bone and articular cartilage were recorded out of a total of 47,222 deaths from cancer [3]. Despite being a rare neoplasm, it presents two bimodal peaks, being the most frequent malignant neoplasm of bone in people under 20 years of age, in which group 50% of patients are diagnosed with osteosarcoma, and presenting another increase in incidence in people over 65 years of age [4]. Therefore, despite being a rare neoplasm, approximately half of the people affected are young, resulting in a high loss of years of potential life in the population. Various risk factors have been described, among which we can highlight exposure to radiotherapy and chemotherapy (Table 1 and Table 2). Regarding this point, osteosarcoma represents the leading cause of solid secondary malignancy in patients who received radiotherapy for a neoplasm in their youth. The time interval to appearance can be up to 20 years, and this aetiology should be suspected in bone tumours in patients with a history of radiation therapy [5]. On the other hand, the chemotherapy that is most often associated with osteosarcoma involves alkylating agents, such as nitrogen mustards or platinum derivatives among the most frequent [6]. We must also point out that, in young people, osteosarcoma is associated with genetic diseases that can account for up to 30% of cases, there having been described a wide variety of genetic conditions that predispose to it [7,8]. Among the hereditary diseases that represent the vast majority of these patients, alterations in Rb1 stand out, which are inherited in a dominant way, and in addition to the characteristic ocular tumour, there is a predisposition to other solid neoplasms where sarcomas represent up to 60% of cases themselves. Similarly, Li–Fraumeni syndrome, which is associated with inherited mutations in p53, is associated with an increased risk of developing osteosarcomas [9]. It is important that the diagnostic criteria for the Li–Fraumeni syndrome include osteosarcoma and other soft tissue sarcomas as the main tumours in this genetic disease [10]. There are other less common hereditary conditions, such as Rothmund–Thomson syndrome, which is associated with various dermatological and ophthalmological alterations and a risk of approximately 30% of presenting with osteosarcoma [11]. Other less common syndromes, such as Bloom syndrome and Werner syndrome, have also been associated with an increased risk of osteosarcoma in children and adults [12]. We have previously noted that there is another peak in incidence in older people. This increased incidence can be associated with various bone diseases, most notably Paget’s disease, which is characterised by an alteration in the bone turnover process and affects up to 1% of the population over 55 years of age in Spain. The probability of developing osteosarcoma in these patients is approximately 1% of patients, and progression to invasive disease usually occurs in bone affected by Paget’s disease, where it is also common for several areas of the bone to be affected at the same time. This leads to more aggressive tumours that are difficult to treat and therefore have a worse prognosis [13]. Various genetic alterations have been associated with the invasive progression of Paget’s disease, relating to alterations in chromosome 18 or aberrant variants in chromosome 5 [14].

From a pathological point of view, the 2020 WHO classification allows the histological differentiation of different grades of osteosarcomas, such as low-grade, periosteal, high-grade, unspecified osteosarcoma with different variants, and secondary osteosarcoma, each with its own frequency and different prognosis [15]. Within non-specified osteosarcoma, conventional osteosarcoma represents more than 90% and usually affects the metaphysis of long bones in the intramedullary region. Other tumours, such as low-grade sarcoma, which accounts for up to 2% of osteosarcomas, and parosteal tumours, have a relatively good prognosis with cure rates of up to 90% with surgical resection [16]. As we have previously indicated, osteosarcomas, probably due to past radiotherapy or Paget’s disease, are grouped in the classification of secondary osteosarcomas. There are other rarer variants with a worse prognosis, such as multifocal sarcoma that can affect various bones synchronously and craniofacial osteosarcoma [17]. Patients in both situations have an ominous prognosis and are usually candidates for palliative chemotherapy, with a fatal outcome and very short survival in most cases. From an anatomical point of view, osteosarcoma is usually located in the metaphysis of long bones in children (mainly the distal femur and proximal tibia) and in the lower limb in adults [18]. The vast majority of patients usually present week-long pain, constitutional syndrome with asthenia or weight loss, and a tumour in the knee region. Given the bone instability, there is a high probability of pathological fracture, which can be one of the main causes of visits to the emergency room, so multidisciplinary management with traumatology is recommended to avoid this situation [19]. Regarding the diagnosis, simple radiography is usually the first test to be performed, and alterations in the trabecular bone pattern, pathological fractures, periodic reaction and ossification of adjacent soft tissue that give a rising-sun image can be observed. Radiologically, the ideal imaging test for assessing both bone breakdown and soft tissue involvement and local infiltration is magnetic resonance imaging. In addition, this test will allow both biopsy planning and possible surgical intervention [20]. The definitive diagnosis is made by guided biopsy, allowing the correct identification of the histological variety, which is important given the differences in prognosis that depend on differences in pathological variety. Both directed and open needle biopsy should be carefully planned, and the field to be biopsied should be properly delimited, since there is the possibility of tumour spread along the insertion path of the needle within the tumour mass [21]. Upon diagnosis, up to 20% of patients have metastatic disease, primarily to the lungs (~80% metastases in these patients) followed by other bones. For this reason, these patients should be evaluated with chest radiography or CT of the chest, abdomen, and pelvis for the evaluation of disseminated systemic disease [22]. Another useful imaging test is PET CT, which can help locate lung or bone metastases. For bone dissemination, bone scanning may be an alternative for localisation [23]. In any case, metabolic dissemination according to the AJCC criteria already implies a stage IV malignancy, where survival is limited and recurrences are early and aggressive in most cases. The long-term prognosis is determined by the spread of the disease. For example, the 5-year survival of localised disease is 77%, while the presence of disseminated disease reduces the 5-year survival to 26% [24]. The prognosis of patients in recent years has improved thanks to the application of different chemotherapy regimens associated with surgery, but given the complexity of managing this disease, patients should be referred to expert centres where a multidisciplinary approach can be implemented [25]. There is currently no standard chemotherapy regimen available, and there is insufficient evidence to compare the survival benefits of preoperative and postoperative chemotherapy. In cases of localised disease, according to the results of the EURAMOS-1 clinical trial, which included 2260 patients, the regimen of choice is based on methotrexate, doxorubicin, and cisplatin, although there is currently no established standard treatment for this type of tumour [26]. On the other hand, the surgical approach depends on the degree of involvement, location, and locoregional invasion and often leads to the total amputation of a limb in those possible cases. In the case of metastatic dissemination rather than resection candidacy, the previously described regimen of methotrexate, doxorubicin and cisplatin may be appropriate, but there are no data to establish therapeutic lines [27]. Therefore, although osteosarcoma is a rare tumour, there are still major questions regarding its management, which applies to young people half of the time and involves cases of great prognostic uncertainty.

**Table 1:** Main biomarkers and translational applications explored in osteosarcoma.

Marker	Translational Applications	Ref.
Lactate dehydrogenase (serological)	Elevated serological levels are associated with a worse prognosis.	[28]
Alkaline phosphatase (serological)	Elevated serological levels are associated with a worse prognosis.	[29]
TIM-3 (serological)	Elevated serological levels are associated with a worse prognosis and allow differentiation between benign bone lesions and osteosarcoma.	[30]
WNT6 (serological)	Elevated serological levels are associated with a worse prognosis and allow differentiation between benign bone lesions and osteosarcoma (AUC 0.854)	[31]
SAA and CXCL4 (serological)	Elevated serological levels are associated with a worse prognosis.	[32]
P53 (genetic)	Mutations in P53 are associated with more aggressive tumours and Li–Fraumeni syndrome.	[33]
Tb1 (genetic)	Mutations in Rb1 are associated with more aggressive tumours.	[34]
NOTCH1 (genetic)	Worse prognosis and higher rate of metastatic disease.	[35]
C-Fos (genetic)	Greater histological aggressiveness and invasion.	[36]
HER2 (genetic)	Limited prognostic utility.	[37]
C-Myc (genetic)	Worse prognosis and more aggressive lesions.	[38]
FGFR1 (genetic)	Worse response to chemotherapy and worse prognosis.	[39]
PTEN (genetic)	It is associated with a better prognosis.	[40]
miR16 upregulation	Less histological invasion and greater response to cisplatin.	[41]
miR21, miR 214, miR 29, miR 9 and miR 148a upregulation	Worse prognosis and worse average survival.	[42]
miR-382, miR26a, miR-126, miR-195 and miR-124 downregulation	Worse prognosis and worse average survival.	[42]
MiR- 205-5p, MiR-214, MiR-335-5p y MiR-574-3p	Diagnostic utility with AUC of 0.70, 0.8 and 0.88, respectively.	[43]
miR 214	Low plasma levels were associated with better median survival.	[43]
Combination of miR-195-5p, miR-199a-3p, miR-320a and miR-374a-5p	AUC of 0.96 in differentiating patients with osteosarcoma versus healthy controls.	[44]
miR-152 downregulation	AUC 0.956—sensitivity of 92.5% and specificity of 96.2% in differentiating osteosarcoma from periostitis and healthy controls.	[45]
miR 326	AUC of 0.817 diagnosing osteosarcoma compared to healthy controls and decreased levels of miR 326 tend to present a worse prognosis and a higher probability of metastatic disease.	[46]
Circulating tumour cells	Worse prognosis and predictive sensitivity to different chemotherapies.	[47,48,49]

**Table 2:** Main risk factors in osteosarcoma.

Genetic risk factor ~30% osteosarcomas	Acquired risk factor ~70% osteosarcomas
Alterations in Rb1 Li–Fraumeni Syndrome Rothmund–Thomson syndrome Bloom syndrome and Werner syndrome	Previous use of alkylating agents, such as nitrogen mustards or platinum derivatives Prior use of radiotherapy Paget disease

## 2. Serological Markers

The role of biomarkers in different tumours is based on their ability to detect, both serologically and histologically, different molecules that have an impact on the diagnosis, prognosis and follow-up of patients with different oncological diseases. The detection of tumour markers such as CA 19-9, CA 125 and CA 15.3 peripherally is such that the higher the level of serological elevation, the greater the tumour burden, the greater the probability of relapse and the worse the prognosis [50,51]. Currently, in osteosarcoma, the diagnosis and evaluation of disseminated disease are based on radiological tests and biopsy. Practically, the only complementary serological test is the detection of elevated levels of alkaline phosphatase and lactate dehydrogenase, which are only elevated in half of the cases and are a consequence of bone turnover without having clear repercussions at a diagnostic, prognostic or follow-up level [52]. Therefore, although in most tumours there are biomarkers that support the diagnosis and have demonstrated their usefulness in the management and follow-up of these patients with osteosarcoma, follow-up is currently based on imaging tests without a recommendation of clear chronological tracking. The detection of tumour antigens as a form of peripheral biomarker is a diagnostic and follow-up standard in numerous neoplasms. In normal clinical practice in osteosarcoma, it is not possible to detect these molecules, although different authors have evaluated the usefulness of different serological markers. Currently, the most commonly used and most controversial are alkaline phosphatase and lactate dehydrogenase. Given the disparate results of different studies in recent years on their usefulness, different authors have evaluated their usefulness in the prognostic diagnosis of osteosarcoma. In reference to lactate dehydrogenase, one of the most relevant studies comes from Fu et al. In a meta-analysis of 18 studies that included 2543 patients with osteosarcoma, it was observed that high levels of LDH in peripheral blood were accompanied by a worse prognosis [28]. Regarding alkaline phosphatase, the meta-analysis by Hao et al. included 12 studies and provided evidence that high levels of alkaline phosphatase were associated with a worse prognosis and worse average survival in patients with osteosarcoma [29]. These results are in line with Sahran et al., where evidence from a study of 163 patients showed that high levels of LDH and ALP were related to worse prognosis, although, after a multivariate analysis, the high levels of LDH are the most clearly related to the prognostic value [53]. Other serological markers, such as TIM-3 (T-cell immunoglobulin domain and mucin domain-3), were studied by several authors. For example, Ge et al. evaluated the diagnostic and prognostic utility of TIM-3 in 120 patients with osteosarcoma, comparing with 120 control subjects and 120 patients with benign bone tumours. Their results not only demonstrated the usefulness of TIM-3 in differentiating osteosarcoma patients from those with or without benign bone lesions but also demonstrated that elevated levels of TIM-3 were associated with poorer median survival and poorer prognosis [30]. On the other hand, Kai et al. described the utility of Wingless-Type MMTV Integration Site Family 6 (WNT6) for diagnosis and follow-up in 88 patients with osteosarcoma compared with 32 patients with Ewing’s sarcoma and 20 patients with osteomyelitis. Their results demonstrate how the detection of peripheral WNT6 mRNA presents an ROC curve with an area under the curve (AUC) to differentiate osteosarcoma from other entities of 0.854 with a sensitivity of 88.4% and a specificity of 77.8%. In addition, elevated levels of peripheral WNT6 are associated with poorer median survival and an increased presence of metastases [31]. Flores evaluated the usefulness of Serum Amyloid A (SAA) and Chemokine Ligand 4 (CXCL4) in 233 patients, where a serological elevation of SAA and low levels of CXCL4 were associated with poorer median survival [32]. We must emphasise that given the rarity of this neoplasm, it limits the possibility of carrying out studies to evaluate the presence of tumour antigen detection in peripheral blood, although possible biomarkers have been shown that can be used both in diagnosis and in allowing the better stratification of those patients with a higher probability of metastatic progression.

## 3. Genetic Markers

Osteosarcomas, like most sarcomas, are characterised by presenting a wide variety of genetic alterations and expressing a highly complex karyotype. The differentiation of genetic alterations based on the age of presentation is characteristic of osteosarcoma since genetic diseases play an important role in paediatric staging. Likewise, numerous driver mutations have been described both in paediatric osteosarcoma, being the one that is most related to genetic diseases, and in adult osteosarcoma, where a great variety of genes are involved, highlighting that up to 30% of osteosarcomas may be due to genetic causes. Among them, we must highlight the deletions of the 3q, 13q, 17p and 18q regions that are mainly related to alterations in the Rb and p53 genes [54]. On the one hand, the Li–Fraumeni disease represents the most frequent cause of genetic disease and is accompanied by mutations in p53 that generate osteosarcoma in up to 12% of carriers of this genetic disease [55,56]. In this case, Chen et al. evaluated the clinical usefulness of alterations in p53, which also represent the most frequent driver mutation of osteosarcoma, being present in up to 90% of cases in a meta-analysis that included 210 patients with a mean age of 26 years and showed worse median survival in patients with p53 mutations [33]. On the other hand, in children, we have retinoblastoma syndrome, with mutations in the Rb1 gene, in which up to 7% of carrier patients are predisposed to develop osteosarcoma [57]. Ren et al. carried out a systematic review that included 12 studies with a total of 491 patients; alterations in Rb1 were associated with higher mortality, a higher risk of metastatic disease and worse response to chemotherapy treatment in patients with osteosarcoma [34]. Another of the genetic diseases significantly associated with osteosarcoma involves alterations in RECQL4, which are mainly associated with Rothmund–Thomson syndrome type II; up to 30% of patients with this condition may present with osteosarcomas [58]. All these associations of osteosarcoma with genetic diseases are important to know because they allow us to know populations with risk diseases that can be subjected to closer surveillance. There are currently no screening programs for osteosarcoma in populations at risk, but given that osteosarcoma can often be confused with benign bone diseases or bone fractures, the correct and early identification of this entity can allow early stages to be detected and provide better long-term prognosis [59]. Among other driver markers, we can find NOTCH1, which has been studied by several authors, including Zhang et al., who showed from immunohistochemistry evaluation in 68 patients that high levels of the marker in osteosarcomas were related to a greater presence of metastasis [35]. We must also highlight the importance of the C-fos gene; authors such as Wang et al. evaluated 54 osteosarcoma cell lines and determined that high levels of Fos were accompanied by lesions with greater histological aggressiveness and invasion [36]. An association between osteosarcoma and HER2 expression levels has also been observed for many years. Although many authors have analysed the usefulness of HER2 as a prognostic factor, Grolick et al. observed in 149 paediatric patients with osteosarcoma that its usefulness as a prognostic factor is not so clear, limiting its usefulness in osteosarcoma [37]. Another one of the most commonly activated oncogenes is c-Myc. The study by Feng et al. allowed us to observe that the activation of c-Myc was accompanied by more invasive lesions in 70 patients with osteosarcoma and that it was associated with a worse prognosis [38]. Various authors have shown that alterations in MyC expression can be found in up to 50% of cases. There are other less frequent alterations, such as FGFR1, which, as demonstrated by Amary et al. in 288 patients, occurs in up to 18.5% of patients and is associated with a worse response to chemotherapy and, therefore, a worse prognosis [39]. On the other hand, there are good prognostic factors, such as PTEN, which was highlighted by Zhou et al. in a review of 13 articles that included 580 patients with osteosarcoma; a positive expression of PTEN was associated with a better prognosis, including a lower incidence of metastasis and larger differentiated tumours, which were, therefore, less aggressive [40]. We have previously noted that there is a difference between osteosarcoma in adults and in children, where the most frequent alterations in both cases are alterations in the expression of the p53 and Rb genes. There are currently no clear guidelines for genotyping these tumours and assessing, based on the expression of different genes, the probability of a more aggressive disease developing, as well as whether to proceed with more aggressive chemotherapy regimens.

## 4. MicroRNA

MicroRNAs are small noncoding RNA molecules of approximately 20 nucleotides that regulate posttranscriptional genes that are related to processes of cell differentiation, proliferation and apoptosis by promoting or suppressing gene expression after transcription. A microRNA molecule regulates the posttranscription of up to 200 different genes, and studying it allows us to understand the underlying pathophysiology of the metastatic process [60]. In relation to osteosarcoma, the implications of microRNAs are multiple and range from maintaining proliferation, promoting metastatic invasion and immunoresistance mechanisms, among others, to overregulating or underregulating oncosuppressive genes or oncogenes that can also be measured in peripheral blood or directly in histological samples through different laboratory techniques; the usefulness of microRNAs lie in them being able to be used not only in diagnosis but also as prognostic factors [61]. In this regard, it has been observed from the histological analysis of osteosarcoma lesions in a study of 40 patients that the overexpression of miR-16 is accompanied by a lower capacity for histological invasion and a greater response to cisplatin [41]. The same happens with other microRNAs such as miR-31, miR-100 or miR-221-3p, miR-29b-1-5p, miR-125b, miR-27, miR-148a, miR-181a-5p, miR-181c-5p, and miR-195, among the many described [62]. In relation to prognostic utility, given the great variety of microRNAs described, we should highlight the systematic review and meta-analysis by Cheng et al., wherein 55 articles were evaluated based on the prognostic utility of different microRNAs. In it, it is evident that an overexpression of miR-21, miR-214, miR-29, miR-9 and miR-148a at the same time as an under-regulation of miR-382, miR-26a, miR-126, miR-195 and miR-124 was associated with worse prognosis and worse mean survival [42]. From a diagnostic point of view, many authors have tried to demonstrate the usefulness of different miRNAs in diagnosis. For example, Allen-Rhoades et al. analysed 30 control patients and 40 patients with osteosarcoma; miR-205-5p had an AUC of 0.70, miR-214 had an AUC of 0.8, miR-335-5p had an AUC of 0.78 and miR-574-3p had an AUC of 0.88 for the diagnosis of this entity; and low plasma levels of miR-214 were accompanied by better median survival [43]. On the other hand, Lian et al. compared the levels of four miRNAs measured in peripheral blood in patients with osteosarcoma and 90 control patients, and the combination of miR-195-5p, miR-199a-3p, miR-320a, and miR-374a-5p had an AUC of 0.96 in differentiating patients with osteosarcoma versus healthy controls [44]. Wang et al. determined that the under-regulation of miRNA 152 allows differentiation with an AUC of 0.956, a sensitivity of 92.5% and a specificity of 96.2% in differentiating patients with osteosarcoma from periostitis patients and healthy controls in in a group of 80 patients with osteosarcoma, 20 with periostitis and 20 healthy controls [45]. In this regard, authors such as Cao et al. have evaluated the usefulness of miR-326, also measured serologically in 60 patients with osteosarcoma versus 20 healthy controls, and obtained ROC curves with an AUC of 0.817; they also observed that patients with decreased levels of miR-326 tended to have a worse prognosis and a higher likelihood of metastatic disease [46]. Given the great variety of microRNAs and their possible diagnostic uses, we should highlight the systematic review by Gally et al. They carried out a systemic review of up to 60 microRNAs in 35 different studies and, given the numerous different studies with various results, were unable to obtain the stratification of a subgroup of microRNAs to be used in diagnosis [63]. This highlights the complexity of having the necessary material; given that the intention is to obtain the peripheral levels of microRNA, complex laboratory techniques are often required that may not be available in many hospitals. Therefore, we can observe that although a great variety of miRNAs are available and their diagnostic and prognostic utility are useful, it is complex to evaluate a set of miRNAs that are appropriate for this entity.

## 5. Circulating Tumour Cells

The concept of circulating tumour cells (CTCs) is based on the existence of epithelial cells in the blood circulatory system derived after a process of angioinvasion and, therefore, metastatic dissemination; these cells are not normally seen in patients without cancer [64]. CTCs are typically found per 10 million peripheral blood leukocytes and are often associated with underlying metastatic disease [65]. It should be noted that the importance of circulating tumour cells has already been described in prostate, breast and colon cancer, where their presence is associated with a worse prognosis and a higher rates of recurrence after chemotherapy or surgery [66]. Multiple methods have been studied for the detection of circulating tumour cells. The preferred method approved by the FDA which is the gold standard is based on the detection of epithelial protein EpCAM and cytokeratins 8, 18 and 19 using the Cellsearch method, which is approved for metastatic breast cancer, prostate adenocarcinoma and colorectal cancer [67]. Other methods for the detection of CTCs, such as the positive immunoselection of EpCAM, negative immunoselection of leukocytes, filtration, immunomagnetic, electrophoresis or flow cytometry, have also shown utility but are not currently approved by the FDA and are based on complex techniques that require very well-trained personnel, which are inaccessible for daily clinical practice [68]. Various authors have shown the prognostic utility of CTCs in osteosarcoma. For example, Wu et al. showed that 93.75% of CTCs were detected in 32 patients with osteosarcoma compared to 10 controls where they were negative; they also showed that patients who maintained high levels of CTCs after surgery and chemotherapy had worse average survival, higher recurrence rates and more metastatic disease [47]. In this regard, Minghui et al. observed that the detection of CTCs in a group of 30 patients with osteosarcomas was related to metastatic disease and worse prognosis [48]. On the other hand, Han et al. evaluated the usefulness of cisplatin nanodeletion in in vivo models of mice with osteosarcoma in a preclinical model, also demonstrating the chemosensitivity of CTCs from 16 patients to cisplatin in these samples [49]. In reference to liquid biopsy by CTC, the main limitations of this technique relate to sample collection and processing techniques, given that CTCs can become fragile and cannot be processed correctly, which can generate false negatives, and entails various diagnostic-therapeutic implications. On the other hand, the mesenchymal origin of sarcomas limits the use of cytokeratins in their detection, requiring the detection of other markers for their correct identification [69]. Authors such as Fasanya et al. have demonstrated by flow cytometry that ganglioside markers 2 and 3, in addition to vimentin, are possible candidates for the detection of osteosarcoma cells in different harvesting techniques with greater superiority compared to EpCAM, which is the classic epithelial cell marker for the detection of circulating tumour cells. In addition, its high price and technical complexity should be noted, as it often requires a support laboratory that not all hospitals can afford [70]. All of this can affect the performance of the diagnosis, decreasing both the sensitivity and specificity in their detection. On the other hand, the low incidence of this disease only generates small groups of patients for evaluating its clinical utility in large clinical trials. Even so, different authors have shown its usefulness in evidencing the presence of metastatic disease, which is one of the main limitations in survival in osteosarcoma.

## 6. Conclusions

**Figure 1:**
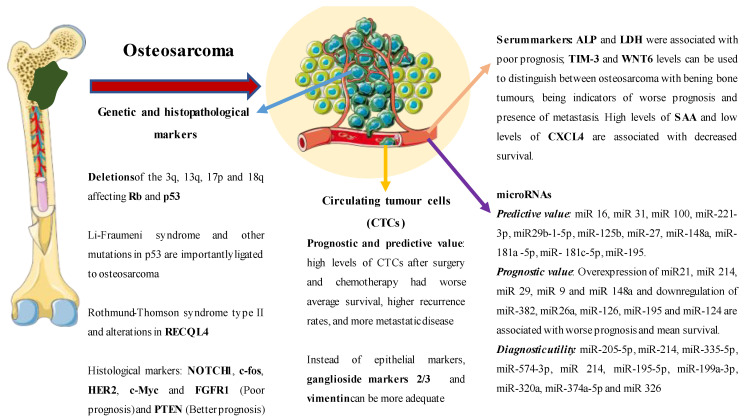
A global perspective on the available biomarkers studied in osteosarcoma.

**Figure 2:**
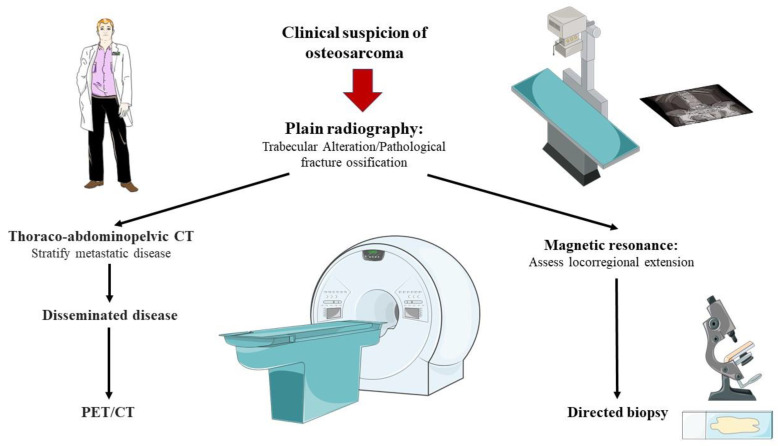
An overview of the clinical diagnosis of osteosarcoma.

Osteosarcoma is a rare cause of neoplasia in developed countries, affecting half of patients with malignant bone neoplasms. Regardless of its frequency, it is a diagnostic challenge requiring a multidisciplinary approach and, in many instances, treatment that cannot limit metastatic disease, presenting these patients with an ominous short-term prognosis. In addition to the disease being aggressive, it should also be noted that chemotherapy treatment in advanced stages does not allow obtaining an adequate response rate and that there is currently no universally accepted chemotherapy regimen available. Although various molecular markers have been described in different neoplasms in recent years, this has not occurred in osteosarcoma (Figure 1 and Figure 2), and practically no targeted therapy has been shown to be clinically useful in these patients. Likewise, the diagnosis of this disease is limited to the use of imaging tests and biopsy, there being very little clinical relevance in the diagnosis and monitoring of different biomarkers. In Table 1, the main applications of the explored biomarkers are summarised Therefore, future objectives in the management of osteosarcoma in its varied histological forms are based on improving early detection and describing new molecular markers in relation to its prognosis and diagnosis that allow the better stratification of those patients with disseminated disease.

## Data Availability

Not applicable.
